# Comparative analysis of serum iron, serum ferritin and red cell folate levels among breast fed, fortified milk and cow’s milk fed infants

**DOI:** 10.12669/pjms.313.6937

**Published:** 2015

**Authors:** Fatima Qudsia, Muhammad Saboor, Shafi Muhammad Khosa, Qamar Ayub, Moinuddin

**Affiliations:** 1 Fatima Qudsia, M.Phil (Hematology). Baqai Institute of Hematology, Baqai Medical University, Karachi, Pakistan; 2 Dr. Muhammad Saboor, Ph.D, MT (ASCP^i^), H (ASCP^i^). Baqai Institute of Hematology, Baqai Medical University, Karachi, Pakistan; 3 Dr. Shafi Muhammad Khosa, MBBS, DCP, M.Phil. Baqai Institute of Hematology, Baqai Medical University, Karachi, Pakistan; 4 Qamar Ayub, Baqai Institute of Hematology, Baqai Medical University, Karachi, Pakistan; 5 Dr. Moinuddin FRCP (C), FRCP (E). Baqai Institute of Hematology, Baqai Medical University, Karachi, Pakistan

**Keywords:** Breast fed, Fortified milk, Cow’s milk, Iron, Ferritin, Folate

## Abstract

**Objective:**

Iron and folic acid are essential nutrients needed for hematopoiesis. Infants’ diet is commonly deficient in these micronutrients that lead to nutritional anemia. Aim of this study was to determine serum iron, serum ferritin and red cell folate levels among healthy breast fed, fortified milk and cow’s milk fed infants.

**Methods:**

A total of 120 infants of 4-9 months of age were enrolled in this study. It included 40 normal breast fed controls, 40 fortified milk fed (FM) and 40 cow’s milk fed (CM) infants. Serum iron, serum ferritin and red cell folate concentrations were determined using colorimetric and enzyme immunoassay techniques.

**Results:**

Mean serum iron, serum ferritin and red cell folate concentrations of breast fed control group were 120.9±68.4µg/dl, 109±71.7ng/ml and 1044.1±409.2ng/ml respectively. Fortified milk (FM) group showed significantly decreased serum iron (p<0.003) as compared with controls whereas serum ferritin and red cell folate values showed insignificant change (p=0.25 and p=0.85 respectively). However serum iron, serum ferritin and red cell folate were significantly decreased in cow’s milk fed (CM) group as compared with control subjects (p<0.04, p<0.006, p<0.02 respectively). Comparison of these biochemical parameters between FM and CM groups showed statistically significant difference of serum ferritin and red cell folate among cow’s milk group (p<0.0001 and p<0.02) whereas serum iron level showed no significant difference, a p-value being 0.38.

**Conclusion:**

Healthy breast fed infants do not need any supplementation and fortification of iron and folic acid. Fortified milk appears to be an acceptable alternative in the absence of breast milk whereas cow’s milk is a poor source of iron and folic acid in infants.

## INTRODUCTION

Anemia is a common health problem in developing countries. World Health Organization has estimated more than 1.5 billion anemic people all over the world. Infants and young children are especially afflicted with this clinical condition. In the developing world about 47% of the young population is found to be anemic. Various factors contribute towards the development of anemia in infants; deficiency of micronutrients is the most important.^1^,^2^ Nutritional anemia is a condition in which the hemoglobin concentration is below the normal level due to deficiency of one or more of the micronutrients required for hemopoiesis.^2^ In Pakistan, 65% of the infants and children suffer from nutritional deficiency anemia.^3^ The most common cause of nutritional anemia is iron deficiency followed by deficiency of folic acid and vitamin B_12_.^2^

Iron deficiency is by far the single most common nutrient deficiency among infants. Worldwide prevalence of iron deficiency in young children is 43%. In Pakistan iron deficiency is a serious health problem affecting more than 80% of the young population.^4^ Iron is an essential element for nearly all cell functions including oxygen delivery, energy production and cell division. Erythropoiesis has inseparable association with iron. Erythrocytes and their precursors require iron for the synthesis of heme which is the central part of hemoglobin.^5^ During rapid growth such as in infancy, adequate iron is crucial for maintaining normal development. Several mechanisms are involved for iron deficiency in infants. Increased iron requirement and physiological occult intestinal bleeding are the major factors in healthy infants. In addition, infants’ diet is particularly deficient in bio available iron.^6^

Folic acid/folate is one of the compulsory micronutrients that are necessary for DNA replication and cell growth. It plays an important role in the proliferation and maturation of erythrocytes. Folic acid deficiency impairs DNA synthesis and causes megaloblastic anemia.^7^ WHO describes folic acid deficiency as a basic health problem in many countries.^8^ In Pakistan 45% of the population with macrocytic anemia has folate deficiency.^9^ Adequate supply of this vitamin during infancy is essential for normal growth and development.^10^ Breast fed infants are most likely to receive sufficient amount of folate. Although folate levels of human milk is simulated in infant formula but data are lacking on folic acid concentrations in fortified milk fed infants. Inadequate food intake, imperfect dietary quality, poor bioavailability and presence of infections are the main reasons for the micronutrient deficiencies among infants.^11^ In growing children micronutrient deficit is responsible for physiological impairment, metabolic disorders and subnormal psychomotor development.^2^

There have been very few studies in this region on nutritional deficiency anemia among infants. Purpose of this study was to evaluate whether breast milk is more nutritious than fortified milk and cow’s milk and to determine which type of feeding is adequate to meet iron and folic acid requirements in 4-9 months’ old infants.

## METHODS

This study was approved by the ethics committee of Baqai Medical University. Written informed consent was obtained from the parents/guardians’ of infants before collection of blood samples. Healthy infants fed on fortified milk (n=40) and cow’s milk (n=40) between 4 to 9 months of age were recruited as test groups. Forty healthy breast fed infants of the same age with normal hematological parameters (Hb> 10.5g/dl, MCV> 75 fl, MCH>25 pg and MCHC>30%) and no history of any recent illness or drug intake were enrolled as controls. Venous blood samples were collected in EDTA and un-anticoagulated tubes. Serum was separated from unanticoagulated tubes and stored at -20ºC. Tests were performed within10 days of collection. Serum iron was determined by colorimetric method using Ferrimat kit (Bio-Merieux France). Serum ferritin was measured using ferritin enzyme immunoassay test kit (DiaMetra, Italy). EDTA anticoagulated samples were collected for red cell folate determination and kept in the refrigerator at 4-6ºC. Tests were performed within two days of blood collection. Measurement of red cell folate was done by Elecsys Folate III competitive immunoassay method.

### Statistical analysis

Results were analyzed using SPSS version 17. Independent t-test was used for data analysis. p value of <0.05 was considered statistically significant.

## RESULTS

Mean serum iron of control group was 120.9±68.4 µg/dl. Fortified milk (FM) and cow’s milk (CM) groups showed significantly decreased serum iron (p<0.003 and p<0.04 respectively) as compared with control group. Mean ± SD of serum ferritin and red cell folate were comparable in FM group and control group with insignificant p values (p=0.25 and p=0.85 respectively). Serum ferritin and red cell folate levels in CM group were considerably decreased as compared with normal controls (p<0.006 and p<0.02), as shown in Table-I.

**Table-I:** Serum iron, serum ferritin and red cell folate levels in 4-9 months old infants of breast milk (BM), fortified milk (FM) and cow’s milk (CM) groups.

Parameters	BM	FM	CM
Serum iron µg/dl	120.9 ± 68.4	77.3 ± 31.0 p=(0.003)	67.3 ± 39.6 p=(0.04)
Serum ferritin ng/ml	109 ± 71.7	137.7 ± 90.6 p=(0.25)	24.0 ± 22.8 p=(0.006)
Red cell folate ng/ml	1044.1 ± 409.2	769 ± 388.6 p=(0.85)	506.4 ± 149.6 p=(0.02)

Data are shown as Mean ± SD. Data shown in parenthesis are p values.

Comparison of these biochemical parameters between FM and CM groups showed statistically significant difference of serum ferritin and red cell folate (137.7±90.6 Vs 24.0±22.8; p<0.001 and 769±388.6 Vs 506.4±149.6; p<0.02) whereas serum iron did not show any significant difference; p-value being 0.38 as shown in Table-II.

**Table-II:** Serum iron, serum ferritin, and red cell folate levels in 4-9 months old infants fed on fortified milk (FM) and cow’s milk (CM)

Parameters	FM	CM	p value
Serum iron µg/dl	77.3 ± 31.0	67.3 ± 39.6	0.38
Serum ferritin ng/ml	137.7 ± 90.6	24.0 ± 22.8	0.001
RBC folate ng/ml	769 ± 388.6	506.4 ± 149.6	0.02

## DISCUSSION

Anemia is one of the most common health problems across the globe. In developing countries approximately half of the infant population is anemic.^12^ Nutritional deficiency anemia is the most common type of childhood anemia. Micronutrients e.g. iron and folic acid have a direct role in hematopoiesis.^13^ Infants’ diet is commonly deficient in these essential nutrients that may affect their physical growth and development.^14^ In this study serum iron, serum ferritin and red cell folate levels were determined in infants fed on breast milk (control), fortified milk (FM) and cow’s milk (CM).

In FM group, mean serum iron was considerably decreased (p<0.003) whereas serum ferritin was comparable to that of control group. These findings strongly support the observations of Dewey et al. who described normal levels of serum iron in breast fed infants. They recommended breast feeding in infants without additional need for iron fortification and supplementation.^15^ Results of Dube et al.^16^ and Monterrosa et al.^17^ are at variance with our findings. They reported iron deficiency in healthy breast fed infants as compared with fortified milk fed group. In another study significantly increased concentration of serum iron was reported among breast fed and fortified milk fed infants. These workers found significant similarity of serum iron among breast fed and iron fortified milk fed infants till 6^th^ months of age.^18^ Raj and his colleagues observed normal serum ferritin in healthy breast fed infants and decreased serum ferritin concentration with age.^19^

Cow’s milk is a poor source of iron in infants. Low iron content and increased excretion of blood in stool are the main factors.^20^ In this study, cow’s milk fed infants were found iron deficient with significantly decreased serum iron and ferritin levels; p<0.04 and p<0.006 respectively as compared with control group. These findings are in conformity with Ziegler^6^ (Thorsdottir^20^ and Oliveira et al.^21^ who also found cow’s milk as an inadequate source of iron. Elalfy et al.^22^ reported similar results of serum ferritin among cow’s milk fed infants. These workers support fortified milk as a better substitute for breast milk.^22^

Folic acid is another essential micronutrient for hematopoiesis. Adequate supply of this vitamin is essential for normal growth and development of healthy infants.^10^ Biochemical parameters to establish folate deficit include red cell folate and serum folate levels. Red cell folate is more reliable than serum folate for accurate assessment of folic acid in milk fed infants.^23^,^24^

In breast fed control group mean± SD of RBC folate concentration was 1044.1 ± 409.2 ng/ml. FM group of infants had comparable results with that of controls. However cow’s milk fed group showed significantly decreased red cell folate as compared with controls (p<0.02).

EK found low concentrations of red cell folate in cow’s milk fed infants. He also reported higher RBC folate values in breast fed control infants than the adult normal ranges.^25^ Smith 1985 and Hay et al. 2008 are not in agreement with this observation and reported cow’s milk as good a source of folate as other milk.^24^,^26^ Agostoni and Turck also agreed with these observations and described cow’s milk as a good nutritive diet for infants; the only exception being low iron content.^27^ Our findings of folate deficiency among cow’s milk fed infants are at variance with these observations.

Fortified milk contains specified amount of folic acid which is similar to that of human milk.^17^ Our studies are in agreement with this statement as comparable results of RBC folate among breast fed controls and FM group were observed.

In the light of the above results and the literature review it is concluded that healthy breast fed infants do not need any supplementation and fortification of iron and folic acid. Iron requirements are fully met till 7th month of age in breast fed infants whereas folic acid remains at upper normal levels till 9th month of age. Folic acid deficiency is thus uncommon among healthy breast fed infants. Fortified milk can be a good source of iron and folic acid but its bioavailability may not be the same in all infants; this should however be confirmed by interventional studies. Cow’s milk is not only a poor source of iron, it also an inadequate source of folic acid in 4-9 months old infants. Studies on other micronutrients like cobalamin, Zinc, Vitamin D and copper in cow’s milk fed infants may further provide additional information of therapeutic importance in physical and intellectual development of infants.
